# Chemical Mediation of Oviposition by *Anopheles* Mosquitoes: a Push-Pull System Driven by Volatiles Associated with Larval Stages

**DOI:** 10.1007/s10886-020-01175-5

**Published:** 2020-04-02

**Authors:** Bruce Schoelitsz, Victor Mwingira, Leonard E. G. Mboera, Hans Beijleveld, Constantianus J. M. Koenraadt, Jeroen Spitzen, Joop J. A. van Loon, Willem Takken

**Affiliations:** 1 Laboratory of Entomology, Wageningen University and Research, P.O. Box 16, 6700 AA, Wageningen, The Netherlands; 2 HAS University of Applied Sciences, Onderwijsboulevard 221, 5223 DE, ‘s-Hertogenbosch, The Netherlands; 3 National Institute for Medical Research, Amani Research Centre, P.O. Box 81, Muheza, Tanzania; 4 SACIDS Foundation for One Health, Sokoine University of Agriculture, P.O. Box 3297, Morogoro, Chuo Kikuu Tanzania; 5 Environmental Technology, Wageningen University & Research, Bornsesteeg 59B, 6708 PD Wageningen, The Netherlands

**Keywords:** *Anopheles coluzzii*, *An. gambiae s.s.*, Malaria, Mosquito, Oviposition, Dimethyldisulfide, Dimethyltrisulfide, Nonane, 2,4-pentanedione, Behavior

## Abstract

The oviposition behavior of mosquitoes is mediated by chemical cues. In the malaria mosquito *Anopheles gambiae*, conspecific larvae produce infochemicals that affect this behavior. Emanations from first instar larvae proved strongly attractive to gravid females, while those from fourth instars caused oviposition deterrence, suggesting that larval developmental stage affected the oviposition choice of the female mosquito.

We examined the nature of these chemicals by headspace collection of emanations of water in which larvae of different stages were developing. Four chemicals with putative effects on oviposition behavior were identified: dimethyldisulfide (DMDS) and dimethyltrisulfide (DMTS) were identified in emanations from water containing fourth instars; nonane and 2,4-pentanedione (2,4-PD) were identified in emanations from water containing both first and fourth instars. Dual-choice oviposition studies with these compounds were done in the laboratory and in semi-field experiments in Tanzania.

In the laboratory, DMDS and DMTS were associated with oviposition-deterrent effects, while results with nonane and 2,4-PD were inconclusive. In further studies DMDS and DMTS evoked egg retention, while with nonane and 2,4-PD 88% and 100% of female mosquitoes, respectively, laid eggs. In dual-choice semi-field trials DMDS and DMTS caused oviposition deterrence, while nonane and 2,4-PD evoked attraction, inducing females to lay more eggs in bowls containing these compounds compared to the controls. We conclude that oviposition of *An. gambiae* is mediated by these four infochemicals associated with conspecific larvae, eliciting either attraction or deterrence. High levels of egg retention occurred when females were exposed to chemicals associated with fourth instar larvae.

**Electronic supplementary material:**

The online version of this article (10.1007/s10886-020-01175-5) contains supplementary material, which is available to authorized users.

## Introduction

Assessment and selection of suitable oviposition habitats is important for the life history of mosquitoes (Bentley and Day 1989). Several studies have shown that the selection of oviposition sites by mosquitoes is influenced by chemicals. *Culex quinquefasciatus* Say, for instance, is known to be attracted to a variety of volatiles from breeding sites, including oviposition pheromones produced by conspecific eggs (Otieno et al. 1988) and compounds originating from organic material such as grass infusions and the compound skatole (Mboera et al. 2000a). It has since been shown that odor blends can be used to manipulate egg-laying females of *Cx. quinquefasciatus* and are therefore suitable for monitoring and control of this species (Mboera et al. 2000b). Several *Aedes* species also use chemical cues originating from microbes to identify oviposition sites (Allan and Kline 1995; Santana et al. 2006; Lindh et al. 2008).

The African malaria mosquito *Anopheles gambiae* Giles sensu stricto (hence referred to as *An. gambiae*) is known to be affected by volatiles from micro-organisms in soil and water of breeding sites (Huang et al. 2006; Sumba et al. 2004) and is attracted by water from natural oviposition sites (Herrera-Varela et al. 2014; Okal et al. 2013; Sumba et al. 2008). In addition, female mosquitoes of this species show an olfactory-based preference for oviposition sites in which larvae of the same regional population of *An. gambiae* have developed (Ogbunugafor and Sumba 2008). Furthermore, gravid females are repelled by emanations from breeding sites in which third and fourth instars were developing (Suh et al. 2016). The repellence caused by larvae on egg-laying behavior of gravid females has also been observed to be affected by the density of larvae (Munga et al. 2006). A low density of young larvae had a positive effect on oviposition, whereas high densities of older larvae negatively affected oviposition (Sumba et al. 2008). More recently, the effects of larval stage and density have been studied in greater detail (Mwingira et al. 2019), suggesting a production of chemical compounds that affect oviposition behavior of conspecific gravid *An. gambiae* females, causing a positive response to cues from first instars and a negative response to cues from fourth instars.

A number of compounds have been shown to attract *Anopheles* mosquitoes to oviposition sites. Recently, Lindh et al. (2015) identified the sesquiterpene alcohol cedrol as an oviposition attractant of *An. gambiae s.s*.. The volatiles of grass species *Echinochloa pyramidalis* and *E. stagnina* were attractive to gravid females of *An. coluzzii* Wilkerson & Coetzee and *An. arabiensis* Patton (Asmare et al. 2017); gravid females of *An. arabiensis* were furthermore attracted to volatiles from maize pollen, including *alpha*-pinene*,* limonene*, p*-cymene, nonanal and benzaldehyde (Wondwosen et al. 2017). Paradoxically, the compounds dimethyl disulphide (DMDS) and trimethyl disulphide (DMTS), products of decaying plant material, were identified as oviposition repellents for *An. coluzzii* (Suh et al. 2016). Additionally, Bermuda grass hay infusions contained olfactory compounds that repelled *An. gambiae* (Eneh et al. 2016a). These studies show that the oviposition behavior of *An. gambiae* s.l. females is affected by olfactory cues, which may be attractive or repellent, but the nature of these compounds is still poorly understood, especially concerning the interactions between water-associated cues and conspecific cues. While these studies have identified several compounds originating from natural breeding sites affecting oviposition, compounds associated with larvae have to-date not been described.

The identification of infochemicals influencing oviposition behavior is important for a better understanding of the chemical ecology of oviposition, manipulation of mosquito oviposition behavior and application in monitoring and control methods (Munga et al. 2006; Sumba et al. 2004; Sumba et al. 2008). The identification of oviposition attractant chemicals is expected to complement the current methods of monitoring and controlling mosquito populations (Dugassa et al. 2016; Perich et al. 2003; Ponnusamy et al. 2015). The present study was carried out to determine the effect of larval stage on attraction and repellence of gravid females of *An. gambiae* in laboratory and semi-field settings and to identify volatile chemicals produced by larvae of this species that mediate this behavior.

## Methods and Materials

Laboratory experiments were conducted at the Laboratory of Entomology of Wageningen University & Research in The Netherlands and at the Amani Research Centre of the National Institute for Medical Research, Muheza, Tanzania. The semi-field study was conducted at the Amani Research Centre in Tanzania.

### Insects and Rearing Procedures

We used *Anopheles coluzzii* originating from Suakoko, Liberia, previously known as *An. gambiae s.s.* M form (Coetzee et al. 2013) that was reared at the Laboratory of Entomology, Wageningen University & Research, The Netherlands. Larvae were raised under standardized conditions (water surface >2 cm^2^ per larva), in a climate-controlled chamber at 28 °C and 80% relative humidity, with a 12:12 h L:D photoperiod. Larvae were reared in 2.5 l plastic trays filled with acclimatized tap water and were fed 0.003 g/larva Tetramin® fish food (Tetra Werke, Germany per day). Pupae were collected daily and placed in small cups inside a 30 × 30 × 30 cm Bugdorm® cage (https://www.shop.bugdorm.com) for emergence. Adults (males and females) were kept in a Bugdorm® cage with ad libitum access to a 6% glucose solution. When 3–5 days old, females were fed blood by offering a human arm. Gravid mosquitoes from this group were used to study response to volatiles produced by larvae in the laboratory. Ethical approval for blood feeding was not requested as this method of blood feeding is not subject to the Dutch Act of Medical Research involving Human Subjects (WMO). In our anopheline mosquito cultures, no experimental infections took place and mosquitoes were free of any parasite.

At the Amani Research Centre*,* adult *An. gambiae s.s.* (originating from Ifakara, southern-central Tanzania) were kept in a 30 × 30 × 30 cm metal framed cage covered with netting. Larvae were reared in round aluminium pans with a diameter of 27 cm, filled with filtered tap water to a depth of 2 cm. Larvae were fed on Tetramin® fish food (Tetra Werke, Germany) and were kept in a 12:12 h L:D light regime. The temperature in the insectarium was 29 °C. Pupae were removed from the trays daily and were placed in the mosquito cages for emergence. Male and female mosquitoes were kept in the same cages. For blood feeding, 3–5 days old females were offered a human arm. An approval involving human subjects in blood feeding mosquitoes was obtained from the Medical Research Coordinating Committee of the National Institute for Medical Research in Tanzania. The same volunteer donated blood to all batches of mosquitoes throughout the study, and mosquitoes were fed blood only once during their lifetime. Gravid mosquitoes from this group were used to study the response to infochemicals in the laboratory and semi-field experiments.

### Oviposition in Response to Larvae

Experiments concerning the oviposition behavior in response to the presence of first or fourth instars were performed at the Laboratory of Entomology in Wageningen. The aim of this experiment was to investigate if the concept that larval habitats of *An. coluzzii* emit chemical cues that mediate oviposition behavior in conspecific adults. Nine-day old female *An. coluzzii* were fed blood on a human arm for ten minutes, two days before the start of the experiment and were kept as described above.

Early-stage larvae (L1) were collected two days after oviposition using a glass pipette. Water drops with larvae were placed on the bottom of a white, dry rearing tray and larvae were counted. Late-stage larval instars (L3/L4) were collected with a plastic pipette (5 ml) and were counted in a similar way as L1 larvae. A total of 100 larvae of the same developmental stage were placed in a plastic oviposition cup (5.25 cm diameter × 3 cm height). The volume of rearing water was removed to a minimum before the transfer of larvae and after the transfer of the larvae cups were filled with tap water to a volume of 30 ml.

**Fig. 1:**
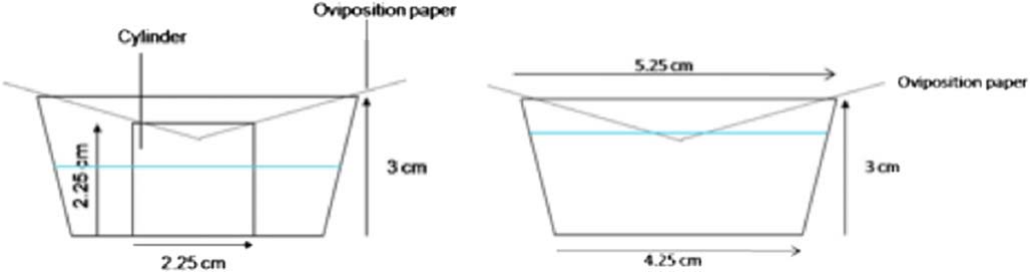
Schematic representation of oviposition cups used in oviposition experiments; showing the set up with larvae (left) and chemicals (right)

Wet filter paper, 125 mm in diameter, Whatman® (Whatman International Ltd., Maidstone, England) was placed over the cup, serving as an oviposition site for the mosquitoes thus preventing stimulation by visual stimuli. To prevent drying out of the oviposition paper, a cylinder made of filter paper was placed in the cups (Fig. 1, left panel). This cylinder ensured that when the water level in the cup decreased, the oviposition paper remained wet. Moreover, because of the cylinder, the oviposition paper did not have to be in contact with the liquid, which would decrease the area of the water surface for the larvae to breathe. Larvae were placed within and outside of the cylinder. As a control the cups were filled with 30 ml tap water.

Gravid mosquitoes were held solitary in a 30 × 30 × 30 cm Bugdorm® cage for 48 h – two oviposition periods (Fritz et al. 2008), under the circumstances described above. Each mosquito was given a choice between ovipositing in a treated cup, with either 100 early-stage larvae (L1) or 100 late-stage larvae (L4), and a control cup. Cups were placed diagonally in corners as far from each other as possible, at a distance of approximately 30 cm. Eggs were counted after 24 and 48 h and the total number of eggs after 48 h was taken as the response of the mosquitoes. Each treatment was repeated 17 times.

### Collection and Identification of Chemicals

Three procedures were conducted at the Laboratory of Entomology, Wageningen University & Research: (i) a proof of concept for emission of volatile chemicals from larval habitats, (ii) collection of volatile chemicals from larval habitats by headspace techniques and (iii) identification of entrapped chemicals by GC-MS.

Volatile compounds released by water containing either no larvae, early stage or late stage larvae of *An. coluzzii* were collected from cups filled with 30 ml of tap water placed in separate air-tight cuvettes.

Volatiles were collected using the “purge and trap” approach on an adsorbing polymer: Tenax-TA 20/35 (Alltech, USA). To reduce background volatiles, air was sucked into the cuvette through a carbon filter and a cartridge containing 100 mg Tenax-TA. Headspace volatiles were trapped at a flow rate of 100 ml/min for 24 h on a second cartridge containing 100 mg Tenax TA connected to the outlet of the cuvette. Samples were released from the adsorbent using a thermodesorption unit (Ultra 50:50 TD, Markes, Llantrisant, UK) while re-collected in an electrically cooled cold trap (Unity, Markes) and followed by gas chromatography (Trace GC Ultra) and mass spectrometry (Trace DSQ quadrupole mass spectrometer), both from Thermo (Thermo Fisher Scientific, Waltham, USA).

The program for thermal desorption consisted of dry purging for 3 min and pre-purging for 1 min using helium (residual oxygen removal) at 30 °C. This was followed by tube desorption at 250 °C for 3 min and the volatiles were focused on a cold trap at 0 °C. Injection onto the analytical column was achieved by heating of the cold trap at the maximum heating acceleration (> 60 °C per second) to 250 °C in a split mode at a split ratio of 1:6. The transfer line between the cold trap and the GC was kept at 160 °C throughout the analysis.

A 30 m × 0.25 mm ID × 1.0 μm F.T. capillary GC column (Rtx-5 MS, Restek, USA) with helium (5.0 grade) as carrier gas at a flow rate of 1.0 mL/min was used for separation of volatile compounds. The GC temperature was programmed as follows: 45 °C for 3 min, followed by a ramp of 8 °C/min to 280 °C and was held at 280 °C for 2 min. The transfer line between the GC and MS was set to 275 °C. MS spectra were recorded by ionization of the column effluent by electron impact (EI) ionization at 70 eV, scanning in positive mode from 35 to 300 *m/z* with a speed of 5 scans per second. The ion source temperature was set to 250 °C and the filament was switched off from 13.6–13.8 min because of a high background peak. Peak identification was performed by comparing the obtained spectra with those in the NIST library (version 2.0 d), experimentally calculated retention indices and using the retention times of authentic synthetic reference compounds.

### Chemicals

The synthetic chemicals dimethyl disulfide (DMDS, ≥ 99.0%), dimethyl trisulfide (DMTS, ≥ 98.0%), nonane (≥ 99.0%) and 2,4-pentanedione (2,4-PD), which is also known as acetylacetone (ReagentPlus®, ≥ 99.0%), all from Sigma Aldrich (Sigma Aldrich, Chemie BV, Zwijndrecht, The Netherlands), were used for testing the oviposition response. Since all of these chemicals were insoluble in water, they were dissolved in methanol and Tween20, in the following ratios: 55 g (test chemical) + 40 ml Methanol +5 ml Tween20. Hereafter, the chemicals were dissolved and diluted in distilled water to make 1 l of diluted chemicals and dilution process continued until the required concentrations for bioassay was reached. The final concentrations of all chemicals ranged from 10^−7^ to 10^−12^ M.

### Oviposition Bioassays

Identified chemicals were tested for effects on oviposition behavior at the Amani Research Centre, Muheza, Tanzania, using *An. gambiae s.s* mosquitoes (Ifakara strain). Two experiments were conducted: laboratory experiments were performed under the same conditions and with the same materials as was done in Wageningen, with the aim to select and confirm effective doses for each chemical. Semi-field experiments were designed to verify potential attractive/repellent effects of these compounds under natural ambient conditions.

### Dose Response Effects on Oviposition

DMDS, DMTS, 2,4-PD and nonane were each tested at six different doses in a four cups choice set up against controls. Gravid *An. gambiae s.s* (48 h post blood feeding) were placed in a 30 × 30 × 30 cm cage. In each cage cups containing 30 ml of a solution of the chemical in concentrations of 10^−7^ M, 10^−8^ M, 10^−9^ M and control, or in concentrations of 10^−10^ M, 10^−11^ M, 10^−12^ M and control were placed. Each of the four oviposition cups was placed in a corner of the cage. Mosquitoes were given a 6% glucose solution as an additional food source. The determination of the most effective concentration was based on the total number and percentage of eggs found in both control and treated cups after 36 h (two nights).

### Dual Choice Experiments with Selected Doses

Based on the results from the dose-response test, dual choice experiments were performed with single compounds. The following concentrations of single compounds were tested against respective controls:

- DMDS: 10^−7^ M and 10^−9^ M
- DMTS: 10^−9^ M and 10^−11^ M
- 2,4-PD: 10^−10^ M
- nonane: 10^−11^ M

### Determination of Oviposition Activity and Egg Retention

To ascertain the effect of emitted infochemicals as either attractive or repellent, an oviposition activity index (OAI) was calculated using the formula OAI = (Nt-Nc)/(Nt + Nc) (Kramer and Mulla 1979), with Nt = number of eggs laid in the egg cup with larvae or test compound, and Nc = number of eggs oviposited in the cup with control materials. Individual gravid *Anopheles coluzzii* females were exposed to emanations of either 100 first or 100 fourth instars; individual gravid females of *An. gambiae s.s.* were exposed to nonane (10^−11^ M), 2,4-DP (10^−10^ M), DMDS (10^−7^ M) or DMTS (10^−9^ M), respectively. Each treatment was replicated 17 times.

At the end of the dual-choice experiments in the laboratory, females were killed and the status of their ovaries was examined for egg retention by dissection of the ovaries. The abdomen of the female was placed on a glass slide, opened with fine surgical forceps and the ovaries were gently pulled out and placed in a drop of physiological saline. The ovaries were examined at 400x magnification under a dissecting microscope. The number of mature eggs present per female were counted (Takken et al. 2013).

### Semi-Field Oviposition Experiments

**Fig. 2:**
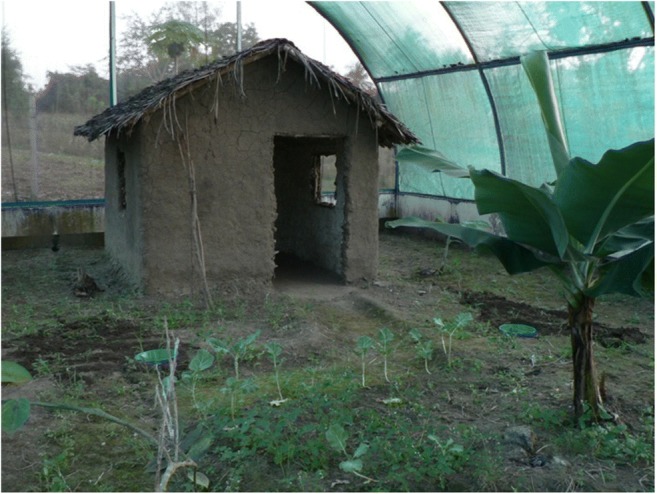
Mosquito sphere at Muheza in which semi-field oviposition studies took place. The sphere had a small house, banana plants, ground vegetation and 2 oviposition bowls in front of the house

The effects of DMTS at a concentration of 10^−11^ M, DMDS at 10^−7^ M, nonane at 10^−11^ M and 2,4-PD at 10^−10^ M on oviposition response were investigated against their controls (distilled water+methanol+Tween20) in a dual choice assay in a semi-field situation (mosquito spheres) at Muheza in Tanzania, under natural ambient conditions (Knols et al. 2002). The objective was to scale up the exploration into a field situation and compare laboratory with semi-field results. Three mosquito spheres (11.4 × 7.1 × 5.0 m) were used in this study (Fig. 2). During the experimental period, the average temperatures in the spheres ranged from a minimum of 16 °C during the night to a maximum of 32 °C during the day. The average relative humidity (RH) ranged from a minimum of 40% to a maximum of 100%.

Two symmetrical holes were dug in the ground at the centre of each sphere, and were located 3 m apart. A green plastic bowl (diameter 26 cm, height 10 cm) was placed in each hole as an artificial breeding site. The bowls were placed in such a way that the rim of the bowls was at ground level. The bowls had a capacity of 5 l and were filled with 3 l of the test solutions of the concentrations mentioned above or with distilled water.

A total of 240 mosquitoes (*An. gambiae s.s*.) were given an opportunity to blood feed twice, on day 3 and day 4 after emergence, and were released on day 5, when eggs had matured (Takken et al. 1998). Mosquitoes were released one hour before dusk (at about 18:00 h), from the centre of the sphere between the bowls. Eggs were counted on the first and second morning after releasing mosquitoes and the solutions were replaced after every experiment. The total number of eggs after two nights was taken as the oviposition response. Each pair in this experiment was replicated 17 times.

### Data Analysis

Differences in oviposition preferences of mosquitoes were analysed using the Wilcoxon matched-pairs signed rank test and Mann-Whitney test for matched-pairs. This non-parametric test was used because the data were not normally distributed. To compare more than two paired groups, like with the dose response test, the Friedman test was used.

Analysis of OAI data was done by comparing the response value with zero. When the OAI values differed significantly from zero with positive or negative values, the treatment was considered to have significant attractant or repellent effect, respectively, on oviposition behavior of gravid females. Oviposition preference of gravid females was determined by OAI values using the Wilcoxon signed rank test (α = 0.05, two-sided). The OAI was also used to compare behavioral assays involving larvae and chemical assays involving identified infochemicals.

The amount of volatiles quantified in headspace collections was analysed using the Kruskal-Wallis test. Differences in egg retention were analysed using the Mann-Whitney U-test.

All tests were performed in SPSS, version 20 (IBM, Armonk, NY, USA).

### Ethical Clearance

The study was conducted according to Standard Operating Procedures approved by the Medical Research Coordinating Committee (MRCC) of the National Institute for Medical Research (NIMR), Tanzania. It received a research permit from MRCC with reference number NIMR/HQ/R.8a/Vol. IX/573 and a permit from the Tanzania Commission for Science and Technology with reference number CST/RCA 138/225/2008. In the Netherlands, ethical approval for blood feeding was not requested as this method of blood feeding is not subject to the Dutch Act of Medical Research involving Human Subjects (WMO).

## Results

### Oviposition in Response to Larvae of Different Development Stages

**Fig. 3:**
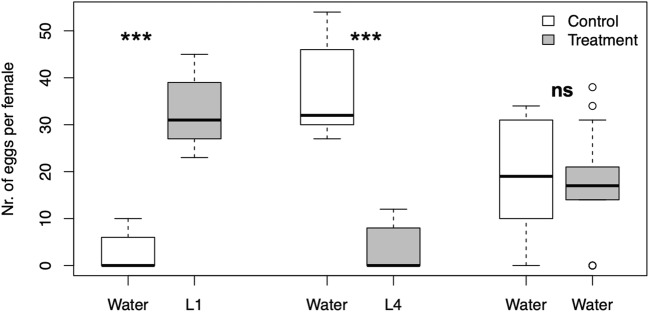
Median number of eggs laid by *An. gambiae s.s.* in a dual-choice test with first and fourth instars. i) cup containing 100 first instars against a control cup, ii) a cup containing 100 fourth instars against a control cup, iii) two cups containing distilled water. Asterisks indicates significance between treatment and control (***, *P* < 0.001, Wilcoxon signed rank test)

Significantly more eggs were deposited in cups containing L1 larvae than in control cups (*P* < 0.0001, Wilcoxon signed rank test), with a median (± SE) of 31.0 ± 2.2 over 0 in control, respectively (Fig. 3). When given a choice between 100 L4 larvae and a control, mosquitoes deposited more eggs in the control cups (*P* < 0.0001, Wilcoxon signed rank test), i.e. median of 0 in the cups with L4 larvae compared to 32.0 ± 3.0 in the control cups (Fig. 3). When two cups with water were tested, there was no difference in the median number of eggs per cup (17.0 ± 3.6 and 19.0 ± 3.8 eggs, respectively).

### Collection and Identification of Infochemicals

**Fig. 4:**
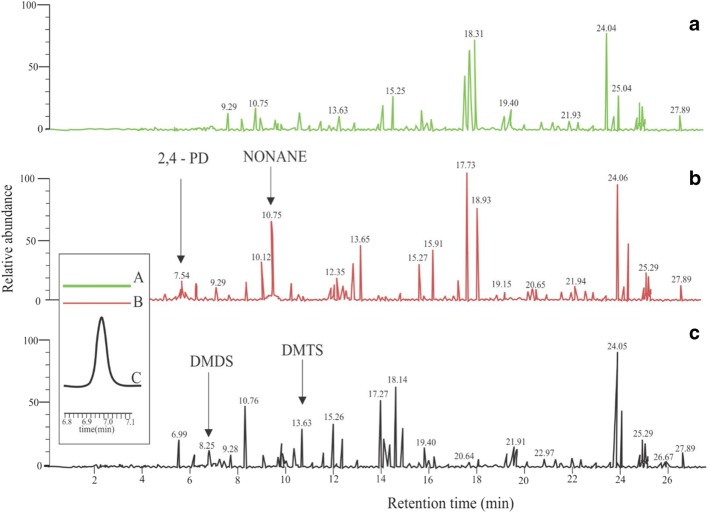
Partial chromatograms for headspace from water containing larvae and control. Volatile samples were entrapped from control water (a) and water with either of two different larval stages, i.e. early stage (L1) (b) and late stage (L4) (c). A mass range zoom (m/z = 94) representing DMDS is added. Y-axis represents equal relative abundances of the different analyses, normalized for the most abundant TIC signal. Peaks for 2,4-pentanedione, nonane, dimethyl disulfide and dimethyl trisulfide are labeled

**Fig. 5:**
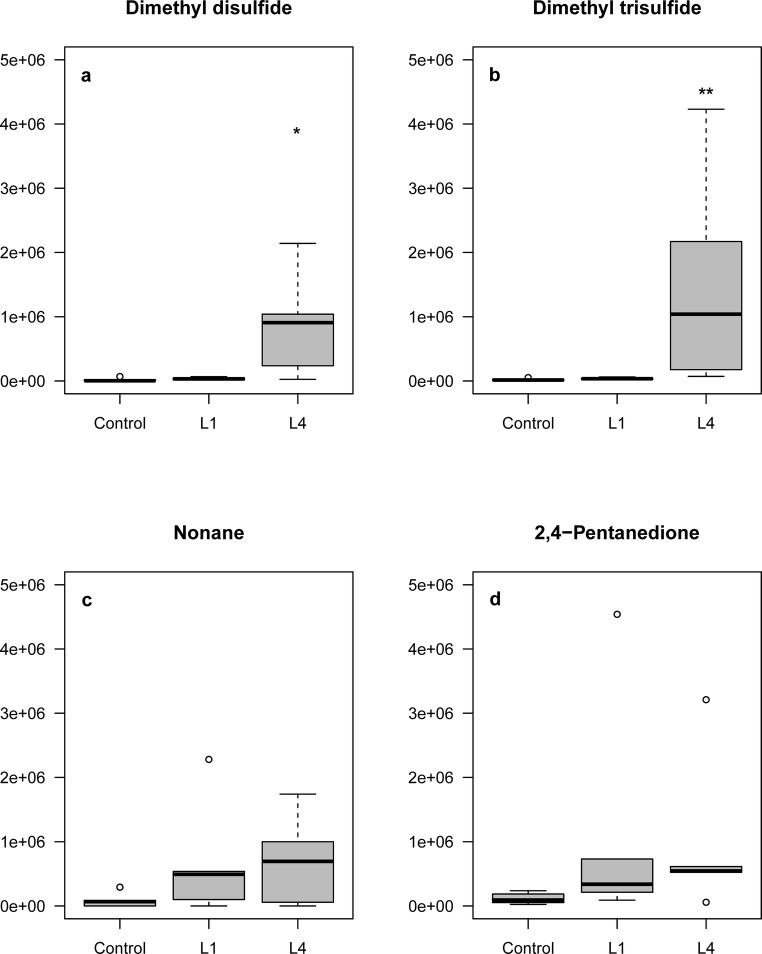
Relative abundance of the selected chemicals in control cups, cups with early stage (L1) and cups with late stage larvae (L4). Graphs show mean abundances of DMDS (a), DMTS (b), nonane (c) and 2,4-PD. Asterisks indicate significant RA value different from control (*, *P* < 0.05, **, P < 0.1, Kruskal Wallis, *N* = 5)

Analysis of the headspace extract from water containing larvae by GC-MS showed that four out of sixteen volatile compounds that showed a difference between control, early stage larvae and late stage larvae were found (Fig. 4). The compounds were identified by matches with database spectra, and by matching their retention times and mass spectra to standards of synthetic compounds. After analysis, only two of these compounds were significantly different in abundance between the treatments. Dimethyl disulfide (DMDS; *P* = 0.021, Kruskal Wallis, *n* = 5) and dimethyl trisulfide (DMTS; *P* = 0.006, Kruskal Wallis, n = 5) were collected in higher amounts from cups containing late-stage larvae than from the control cups or cups containing early-stage larvae (Fig. 5a, b). Nonane (*P* = 0.275, Kruskal Wallis, n = 5) and 2,4-PD (*P* = 0.081, Kruskal Wallis, n = 5) were equally abundant in the headspace from early-stage and late-stage larvae but differed from the control (*P* < 0.05). There was no significant difference in abundance of nonane and 2,4-PD between the cups containing early-stage and late-stage larvae (Fig. 5c, d). These four compounds were selected as putative chemicals influencing oviposition because of their marked greater abundance compared to control.

### Dose Response Effects

No significant differences were found in the number of eggs between the different concentrations and control (*P* > 0.05, Friedman test, N _[all chemicals tested]_ = 12). The concentration of DMDS that received fewest eggs was 5.5 × 10^−7^ M with an Oviposition Activity Index (OAI) = −0.78, average percentage ± SE of 6.5 ± 6.46% of the total number of eggs oviposited and was selected as concentration used in the follow-up experiments. In the second set of concentrations of DMTS (ranging from 5.5 × 10^−10^ – 5.5 × 10^−12^ M) mosquitoes in two of the three cages did not oviposit at all. At the concentration 5.5 × 10^−11^ M, oviposition bowls received fewest eggs with an OAI equal to −0.17, and this concentration was used for further experiments (Fig. S1).

Differences in oviposition response between the tested concentrations of nonane were small for both ranges and the concentration of*.* 5.5 × 10^−11^ M with an average percentage of 38.9, mean 72.7 ± 40.3, OAI = +0.21 was selected for further study. When testing 2,4-PD, the concentration of 5.5 × 10^−10^ M received most eggs, with an average percentage of 47.4, mean 52.3 ± 28.3 and OAI of +0.42. These concentrations were selected for further experiments.

### Dual Choice Experiments in the Laboratory with Selected Chemicals

In the laboratory in Muheza, differences in number of eggs laid between treated and control cups were not significant for any treatment (Table 1, Wilcoxon signed rank test). Only 47% and 44% of the mosquitoes exposed to DMDS and DMTS, respectively, developed eggs and oviposited compared to 100% in the control. The remaining mosquitoes of the DMDS and DMTS treatments had fully developed eggs, but did not oviposit. As a result, the number of eggs that were retained by mosquitoes exposed to the methylsulfides were significantly higher than of mosquitoes in cages with water only (*P* = 0.018 for DMDS and *P* = 0.007 for DMTS, Mann-Whitney U). For nonane, 2,4-PD and the control experiments, the percentages of mosquitoes that oviposited were 100%, 88% and 100%, respectively. The number of eggs retained by mosquitoes exposed to nonane and 2,4-PD were not different from the control.

**Table 1:** Results of oviposition response and examination for egg retention of female *An. gambiae s.s.* exposed to selected concentrations of DMDS, DMTS, nonane and 2,4-pentanedione in a dual-choice set up against distilled water

Treatment	Dose	N	Mean no. ± SE of eggs per female			% females that oviposited	Mean no. ± SE retained eggs per female**
			Treatment	Control	P*		
DMDS	5.5*10^−7^	17	13.41 ± 5.66	18.12 ± 7.51	0.647	47	52.06 ± 14.43 b
DMTS	5.5*10^−11^	16	4.69 ± 2.48	8.88 ± 5.45	0.799	44	37.13 ± 9.02 b
Nonane	5.5*10^−11^	17	24.00 ± 10.27	47.29 ± 11.29	0.147	88	15.90 ± 10.95 a
2,4-Pentanedione	5.5*10^−10^	14	32.86 ± 10.32	36.43 ± 9.47	0.861	100	6.92 ± 6.92 a
Water (=control)		10	48.30 ± 15.59	19.90 ± 9.62	0.241	100	2.20 ± 2.20 a

N = number of replicates with one female per dual-choice test

*Wilcoxon signed rank test

**Differences in letters behind each value indicate a significant difference between the mean number of of retained eggs per female (*P* = 0.018 for DMDS and *P* = 0.007 for DMTS, Mann-Whitney U-test)

### Semi-Field Experiment

**Fig. 6:**
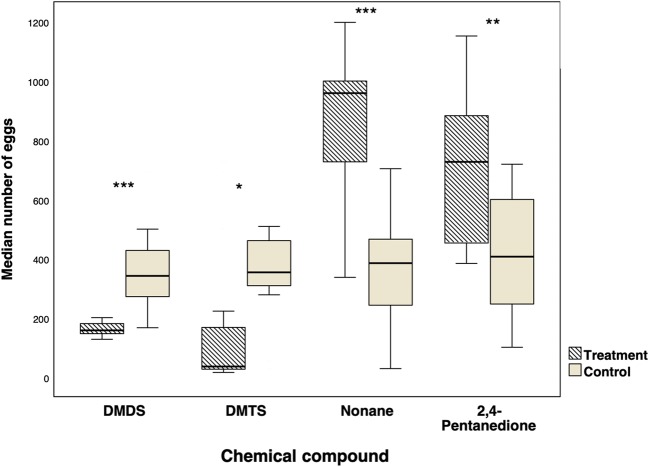
Oviposition response of *Anopheles gambiae,* expressed as the median number of eggs laid per female, when tested in a dual-choice essay with dimethyl disulfate (DMDS), dimethyl trisulfate (DMTS), nonane or 2,4-pentanedione against distilled water. Asterisks indicate significant differences in number of eggs in treatment versus control (*, P < 0.05, **, P < 0.001, ***, *P* < 0.0001; Wilcoxon signed rank test)

There were marked differences in the oviposition effects of DMTS and DMDS on the one hand, and nonane and 2,4-PD on the other hand (Fig. 6). Bowls treated with DMDS or DMTS received significantly fewer eggs than the controls (for DMDS: *P* < 0.0001, *n* = 17, Median _[DMDS]_ = 154; Median _[control]_ = 341; for DMTS: *P* = 0.049, n = 17, Median _[DMTS]_ = 35; Median _[control]_ = 353. The bowl treated with nonane received significantly more eggs than the respective controls (*P* < 0.0001, n = 17, Median _[nonane]_ = 958; Median _[control]_ = 384. Likewise, the bowl treated with 2,4-PD received significantly more eggs than the respective controls (*P* < 0.001, n = 17, Median _[2, 4-PD]_ = 726; Median _[control]_ = 406. The oviposition response to control treatments was similar between the different experiments, indicating consistence in results between different experiments (Fig. 6). The OAIs for nonane and 2,4-PD were positive, indicating stimulation of oviposition activity whereas those for DMDS and DMTS were negative, indicating inhibition of oviposition activity in the presence of these infochemicals.

### Oviposition Activity with Live Larvae and Selected Chemicals

**Fig. 7:**
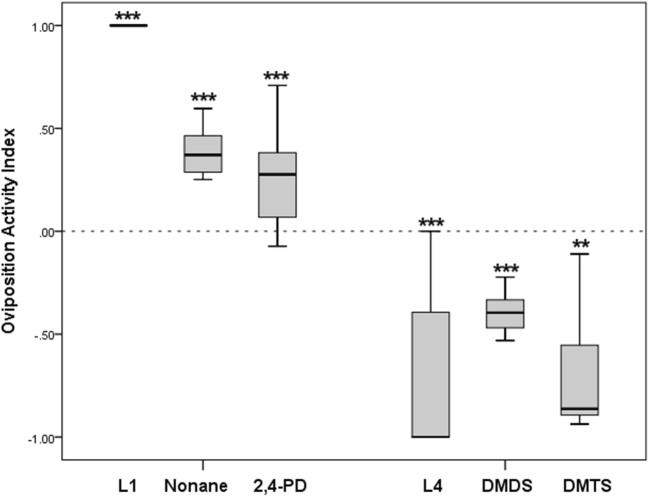
Oviposition activity index (OAI) of gravid *Anopheles coluzzii* exposed to first and fourth instars, and gravid *Anopheles gambiae s.s.* exposed to nonane, 2,4-pentanedione, dimethyl disulfate or dimethyl trisulfate tested in a dual-choice assay. Asterisks indicate an OAI value significantly different from zero (**, P < 0.001, ***, P < 0.0001; Wilcoxon signed rank test)

The oviposition activity in the presence of early and late stage larvae was compared to oviposition activity in the presence of infochemicals entrapped from early and late stage larvae. The oviposition activities of mosquitoes followed a similar trend in response to L1, nonane and 2,4 PD with a positive value that indicates stimulation of oviposition activities (Fig. 7). The median oviposition activity was highest with L1 (OAI = 1.00 ± 0.06, n = 17, *P* < 0.0001) followed by nonane (OAI = 0.36 ± 0.09, n = 17, *P* < 0.0001) and then 2,4-PD (OAI = 0.28 ± 0.07, n = 17, *P* < 0.001). Also, the oviposition activities in response to L4, DMDS and DMTS followed a similar trend, with a negative value suggesting inhibition of oviposition activities (Fig. 7). Fourth instars had the lowest OAI values, (median OAI = −1.00 ± 0.06, n = 17, *P* < 0.0001) followed by DMTS (median OAI = −0.85 ± 0.19, n = 17, *P* = 0.0001) and DMDS (median OAI = −0.4 ± 0.03, n = 17, *P* < 0.0001).

## Discussion

Oviposition behavior of *An. gambiae* females is affected by volatile chemicals associated with conspecific larvae, where first instars affect oviposition positively, and fourth instars cause deterrence and even inhibition of oviposition. Nonane and 2,4-PD are putative attractants, and in a semi-field setting water bodies containing nonane or 2,4-PD received significantly more eggs than untreated water. DMDS and DMTS acted as oviposition repellents and caused egg retention. The data suggest that the oviposition response of these anophelines is mediated by infochemicals associated with conspecifics and that instar stage has a strong impact on this behavior.

Several studies have shown the role of breeding-site specific chemical volatiles affecting oviposition behavior of different members of the *An. gambiae* complex, apart from the role of water vapour as a general cue for all mosquito species (Okal et al. 2013). Indole has been identified to act as an attractant for *An. gambiae*, originating from larval water (Blackwell and Johnson 2000). Lindh et al. (2008) identified 13 putative oviposition attractants derived from bacteria in breeding water. More recently, cedrol was identified as an oviposition attractant for *An. gambiae s.s*. (Lindh et al. 2015). The compound was associated with water derived from a natural breeding site and shown to be the product of rhizomes of the grass *Cyperus rotundus* (Eneh et al. 2016b). This finding is in line with other studies which also showed a strong association of breeding site water, including infusions of plants growing in the water, and oviposition attraction of various members of the *An. gambiae* complex (Herrera-Varela et al. 2014; Asmare et al. 2017). Other identified chemical cues mediating oviposition behavior in *An. coluzzii* mosquitoes include 2-propylphenol and 4-methylcyclohexanol (Rinker et al. 2013). Recently, DMDS, DMTS and 6-methyl-5-hepten-2-one (sulcatone) were identified from headspace analyses derived from habitats that repelled *An. coluzzii* (Suh et al. 2016) and were considered as putative oviposition repellents. This is corroborated by the findings of our study, where DMDS and DMTS caused a significant reduction in oviposition response.

Insects have evolved a wide range of hydrocarbons to protect against dehydration, which had the potential to become signalling molecules involved in communication. Therefore, most insects produce straight chain n-alkanes (Gibbs 1998) which may serve as water-proofing agent, communication and signalling compound (Hölldobler and Wilson 2009). For a hydrocarbon to act as an infochemical from a distance, it must be volatile (Drijfhout et al. 2009), and nonane (C_9_H_20_) fulfils this criterion. Across the range of environmental temperatures at which mosquitoes are active, nonane is a hydrocarbon which volatilizes easily, hence apt to convey information on suitability of a breeding site to gravid mosquitoes.

Behavioral effects of nonane and 2,4-PD on insects have not previously been reported to our knowledge. Recently, the attraction of gravid female *An. arabiensis* to sugarcane pollen was described and the mosquitoes expressed a positive response to headspace extracts of pollen. Among many headspace volatiles of sugarcane pollen, nonane was identified, but the compound did not elicit an EAG response in *An. arabiensis* (Wondwosen 2016). Nonane has been described to show a slightly increased emission by glass beads contaminated with odors of a person less attractive to the yellow-fever mosquito *Aedes aegypti* (L.) than by a more attractive person. However, hydrocarbons generally contribute little to the overall attraction of host-seeking females of this species (Bernier et al. 2002) and a bioactive role of nonane in mosquito host seeking remained unconfirmed.

The OAI of the diketone 2,4-pentanedione is similar to the results obtained with various ketones studied earlier in different mosquito species (Knight and Corbet 1991). The ketones generally cause a positive ovipositional response (Ganesan et al. 2006). Moreover, the diketone 2,4-pentanedione is liable to keto-enol tautomerism, which is a process of migration of an atom within the same organic molecule, leading to a change in its structural skeleton, electron density distribution and chemical properties. 2,4-PD undergoes prototropic tautomerism and exists in equilibrium with its enol tautomer and differs just in the location of a double bond and a hydrogen atom (proton) which often migrates. Tautomers are the chameleons of chemistry, capable of changing by a simple change of phase from an apparent established structure to another and then back again when the original conditions are restored (Antonov 2013). Tautomers are interesting because their optical properties make them suitable as signalling molecules in sensors as they can rapidly switch between states. Many biologically important molecules have several tautomers, among which attractants which are used for luring insects (Pickett 1990). Our finding that in a semi-field setup nonane and 2,4-PD elicited high oviposition activity suggests that both compounds may be used for mosquito surveillance and/or control, as odor baits in oviposition traps (Mboera et al. 2000b; Dugassa et al. 2016; Li et al. 2016).

In our study, both DMDS and DMTS had a negative effect on oviposition of *An. gambiae s.s.* and were collected only from the headspace of fourth larval instars, whereas nonane and 2,4-DP were found in the headspace of both first and fourth instars. Most mosquitoes did not oviposit when DMDS or DMTS were present in the cages, neither in the treated cup nor in the control cup. However, the possibility of saturation of air by these chemicals should not be ignored – as the size of the cage may have had an effect on this outcome. DMDS and DMTS are emitted by a broad range of natural sources; both are produced by bacteria (Khoga et al. 2002). DMDS can be found in human feces (Moore et al. 1987) and both compounds are known to be emitted by plants (Du and Millar 1999; Stensmyr et al. 2002; Soler et al. 2007). Insecticidal and repelling properties of both DMDS and DMTS have been previously described. DMDS has been shown to be an effective insecticide against termites (Dugravot et al. 2002) and cockroach species (Dugravot et al. 2003).

Our observation that DMDS and DMTS both in the laboratory and semi-field caused strongly reduced oviposition, confirms work by others who reported oviposition deterrence in the presence of these compounds (Suh et al. 2016). Like in our study, Suh et al. (2016) studied the effect of suboptimal larval habitats of *An. coluzzii* in a laboratory bioassay and identified DMDS and DMTS in the headspace of water that had been pre-conditioned with late-stage larvae. However, here we show that these compounds also induce deterrence in the semi-field, suggesting the important role of these compounds in natural ecosystems. Sulcatone was also identified in the headspace collections by Suh et al. (2016) and shown to cause oviposition deterrence. Unlike DMDS and DMTS, in the present study sulcatone was not identified to be significantly associated with the presence of mosquito larvae. The difference in results between Suh et al. (2016) and our study is likely to be due to different rearing conditions. It is interesting that capitate peg sensilla of *An. coluzzii* were activated when exposed to DMDS, DMTS and sulcatone, providing physiological indications that the oviposition deterrence is mediated by the olfactory system (Suh et al. 2016).

With the exception of 2,4-PD, optimal dose ranges for an effect on oviposition were lower than 10^−10^ M. For nonane, a dose of 10^−7^ M produced a lower oviposition response, while that of 5.5 × 10^−9^M was highly attractive. The dose ranges of DMDS and DMTS with most effect on oviposition were between 10^−7^ M and 10^−9^ M, which was in the same range as reported by Suh et al. (2016). The dose-response results demonstrate that testing doses over a wide range is crucial for assessing the potential impact on behavior.

The results of the dual-choice tests with the individual chemicals in the laboratory did not match those from the semi field, where nonane and 2,4-DP elicited high oviposition, and DMDS and DMTS suppressed oviposition. As the results with the controls in the laboratory study were highly skewed, and the experimental sets produced outcomes with high standard errors, it is possible that positioning of the experimental cages may have caused a bias in the results. As most females exposed to nonane and 2,4-DP laid eggs, similar to the controls, and females exposed to DMDS and DMTS expressed high egg retention, we conclude that the tested chemicals affected the oviposition behavior in the same way as observed in the semi field study.

The presence of the oviposition attractants nonane and 2,4-DP in the headspace of water bodies containing both larval stages, and the repellents DMDS and DMTS in those containing older larvae only, suggests that the positive effect of compounds emitted by first instars is masked by DMDS and DMTS in fourth instars. The masking effect of chemical compounds has been suggested in host-seeking *Ae. aegypti* females by Logan et al. (2008), and for *An. gambiae* (including both siblings *An. coluzzii* and *An. gambiae s.s.*) serves as a mechanism to prevent oviposition where late-stage larvae are present. Given the close genetic relationship between the members of the *An. gambiae* complex, the data suggest that these oviposition-mediating chemicals are present in the entire complex.

Our results show that oviposition by *An. gambiae* is influenced by chemical compounds associated with conspecific larvae and that the oviposition response is dependent on the stage of the larvae present in the oviposition site. The behavior of mosquitoes in response to larvae present in oviposition sites is consistent with the behavior described in our earlier study (Mwingira et al. 2019). Early-stage larvae attract gravid mosquitoes that oviposit, whereas late-stage larvae repel them, and both behaviors are mediated by infochemicals, nonane and 2,4-DP as oviposition stimulants, and DMDS and DMTS as repellents. This phenomenon may affect larval site selection strategies within mosquito populations and could have an important biological effect on mosquito populations such as competition between species (Koenraadt and Takken 2003). It has been found that larvae of different females of *An. gambiae* were sharing the same habitats, suggesting aggregation by different parent mosquitoes (Chen et al. 2006; Chen et al. 2008). Oviposition sites contain a spectrum of factors influencing oviposition behavior like water type (Sumba et al. 2008), food quality and quantity (Munga et al. 2006). The role of conspecific larvae and other biotic or abiotic factors in oviposition site selection needs to be further explored. Evasion of habitats with L4 larvae by gravid females in the field has not been reported to date.

In conclusion, our results indicate that the attractive effects of chemicals associated with early-stage larvae are cancelled out by the chemicals that are associated with late-stage larvae, presumably DMDS and DMTS. Nonane and 2,4-DP, identified in the headspace of anopheline larvae, elicited a strong oviposition response under semi-field conditions. To our knowledge, this is the first report of identified oviposition attractants associated with anopheline larvae in-vivo. Our observation that conspecific larvae and chemicals associated with them mediate oviposition behavior warrants further studies, especially under field situations. Compounds with a positive effect on breeding-site selection are interesting as potential candidates for applications in ovitraps (Paz-Soldan et al. 2016). The combined use of nonane and/or 2,4-PD and DMDS and/or DMTS in traps can provide a push-pull system, in which mosquitoes are repelled by DMDS and/or DMTS volatilized from dispensers placed in the vicinity of houses and attracted by nonane and/or 2,4-PD, applied in traps positioned just outside of villages.

## Electronic supplementary material

ESM 1 (PNG 120 kb)
