# Supplementary figures and images for: Chemical Mediation of Oviposition by Anopheles Mosquitoes: a Push-Pull System Driven by Volatiles Associated with Larval Stages
Source: J Chem Ecol. 2020 Apr 2;46(4):397–409. doi: 10.1007/s10886-020-01175-5 (PMC7205850; doi:10.1007/s10886-020-01175-5)

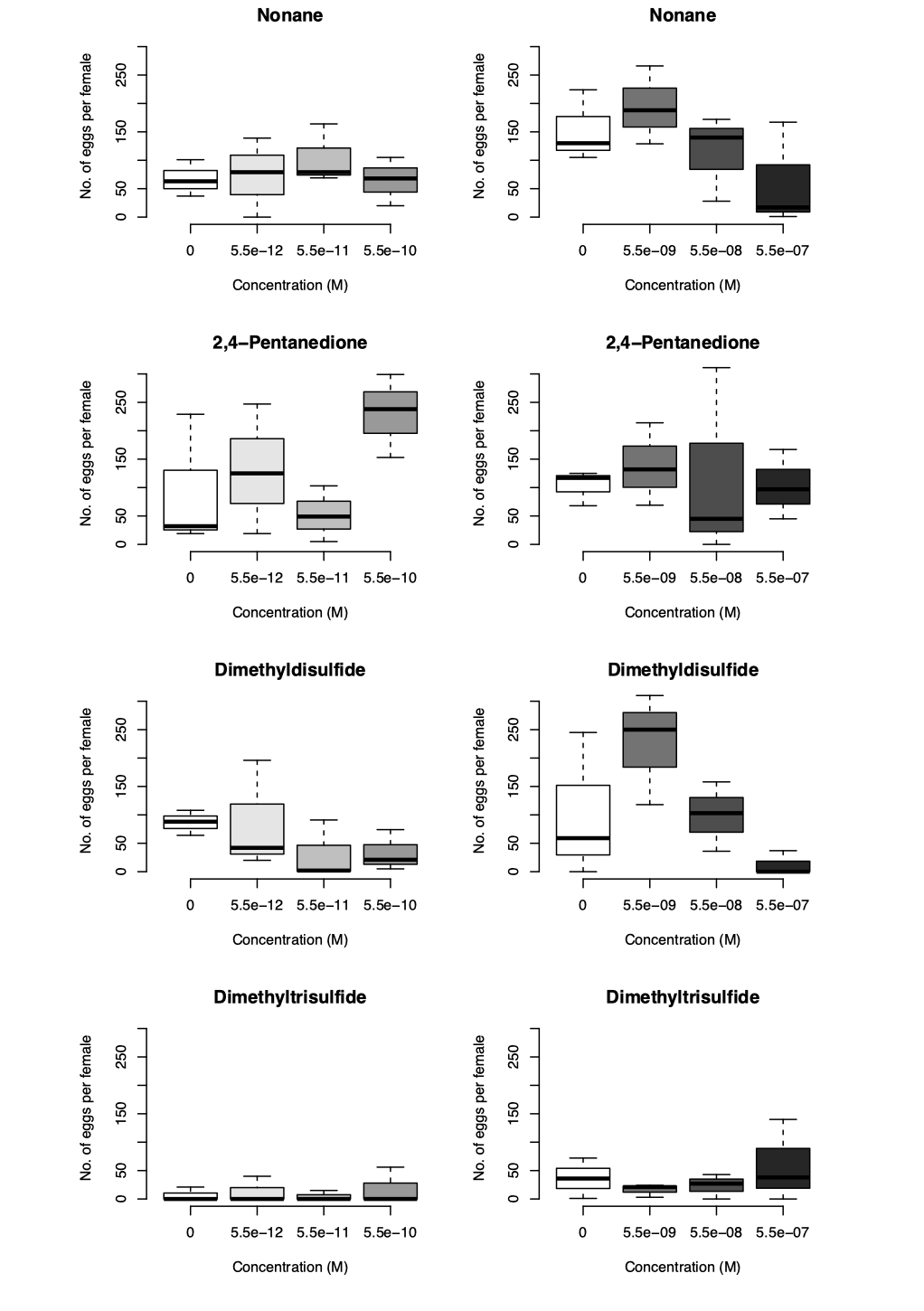

Supplement: Supplementary file 1 — (PNG 120 kb) [file 10886_2020_1175_MOESM1_ESM.png]
